# Learning genotype–phenotype associations from gaps in multi-species sequence alignments

**DOI:** 10.1093/bib/bbaf022

**Published:** 2025-02-20

**Authors:** Uwaise Ibna Islam, Andre Luiz Campelo dos Santos, Ria Kanjilal, Raquel Assis

**Affiliations:** Department of Electrical Engineering and Computer Science, Florida Atlantic University, Boca Raton, FL 33431, United States; Department of Electrical Engineering and Computer Science, Florida Atlantic University, Boca Raton, FL 33431, United States; Department of Electrical Engineering and Computer Science, Florida Atlantic University, Boca Raton, FL 33431, United States; Department of Electrical Engineering and Computer Science, Florida Atlantic University, Boca Raton, FL 33431, United States; Institute for Human Health and Disease Intervention, Florida Atlantic University, Boca Raton, FL 33431, United States

**Keywords:** phenotype prediction, neural network, gene loss, deletion, alignment gap, *Gulo*

## Abstract

Understanding the genetic basis of phenotypic variation is fundamental to biology. Here we introduce GAP, a novel machine learning framework for predicting binary phenotypes from gaps in multi-species sequence alignments. GAP employs a neural network to predict the presence or absence of phenotypes solely from alignment gaps, contrasting with existing tools that require additional and often inaccessible input data. GAP can be applied to three distinct problems: predicting phenotypes in species from known associated genomic regions, pinpointing positions within such regions that are important for predicting phenotypes, and extracting sets of candidate regions associated with phenotypes. We showcase the utility of GAP by exploiting the well-known association between the L-gulonolactone oxidase (*Gulo*) gene and vitamin C synthesis, demonstrating its perfect prediction accuracy in 34 vertebrates. This exceptional performance also applies more generally, with GAP achieving high accuracy and power on a large simulated dataset. Moreover, predictions of vitamin C synthesis in species with unknown status mirror their phylogenetic relationships, and positions with high predictive importance are consistent with those identified by previous studies. Last, a genome-wide application of GAP identifies many additional genes that may be associated with vitamin C synthesis, and analysis of these candidates uncovers functional enrichment for immunity, a widely recognized role of vitamin C. Hence, GAP represents a simple yet useful tool for predicting genotype–phenotype associations and addressing diverse evolutionary questions from data available in a broad range of study systems.

## Introduction

Mapping genotypes to phenotypes is an important and longstanding problem in evolutionary biology [1–5]. Knowledge of the topology of the genotype–phenotype map can inform the structure of the fitness landscape [6], elucidating the effects of mutations and their roles and trajectories in evolutionary and disease processes [7–10]. However, though it remains the crux of our understanding of biology, numerous obstacles stand in the way of resolving the genotype–phenotype map. For one, even determining the function of a single gene requires exhaustive studies of its many properties, including its sequence and impacts of different types of mutations, regulation and expression across diverse conditions, and protein structure and interactions [11]. Additionally, many genes are pleiotropic and influence multiple phenotypes [12–14] and, conversely, many phenotypes are polygenic and controlled by multiple genes [15, 16]. These issues are further compounded by the passage of time, which has eroded, and in some cases erased, signatures of past evolutionary events [17].

Despite these challenges, numerous approaches have been employed to disentangle relationships between genotypes and phenotypes [5, 18–35]. Due to limitations in genetic data, early studies focused on families by utilizing linkage analysis to detect relationships between chromosomal segments and specific phenotypes [18, 22, 23, 29]. With the availability of large genetic datasets from many individuals, genome-wide association studies (GWAS) gained widespread popularity for predicting associations between genetic variants and phenotypes in large populations [5, 20]. At the cross-species level, one can also exploit the evolutionary relationship between sequence conservation and function, enabling detection of genotype–phenotype associations through comparative sequence analysis [19, 26]. More recently, researchers have turned to machine learning to tackle this problem [24, 25, 27, 28, 30, 35–38], as it is naturally suited to making predictions from complex data with correlated or conflicting signals [39]. Additionally, machine learning approaches optimize model fit to training data without necessitating knowledge of the processes that generated the data [39], making them advantageous in scenarios for which the evolutionary model is unknown.

Machine learning algorithms have been increasingly employed to address a diversity of complex predictive challenges in biological research [35–38, 40–42]. Whereas several machine learning methods exist for predicting associations between genotypes and phenotypes [21, 31–38], most of these approaches utilize population-genomic data [31, 32, 34–37], which are not publicly available for the majority of species. In contrast, whole-genome sequence data have been released for over 10,000 species [43]. Additionally, existing tools that make use of genomic data from multiple species also incorporate other heterogeneous sources of information, such as literature searches [21] gene expression patterns and functions [33], or environmental factors [38]. Thus, such methods are not feasible for researchers who only have access to genome sequence data. Last, we were motivated by the idea of developing a tool that could be employed by researchers without extensive computational backgrounds, as multi-species sequence alignments are readily available for numerous taxonomic groups [44, 45]. Hence, we sought to design an easy-to-use machine learning tool that could predict genotype–phenotype associations solely from multi-species sequence alignments.

Here we considered applying a machine learning framework to the simplest scenario, in which a researcher is interested in predicting a binary phenotype, i.e., the presence or absence of a particular trait. Because insertions and deletions commonly cause gains or losses of functions [46–48], we focused on the problem of predicting a binary phenotype from gaps in a multi-species sequence alignment. The goal may be to use an alignment from a single genomic region known to be associated with a phenotype to predict the phenotype in one or more species in the alignment, to use the alignment from this region to predict which positions are associated with the phenotype, or to use alignments from multiple regions across the genome to predict which unknown genomic regions are associated with the phenotype. This task was inspired by [49], who developed a forward genomics comparative approach to predict the association between a gene and phenotype from divergence levels between each species in a multi-species sequence alignment and their reconstructed common ancestor. As a proof-of-concept, [49] showed that their method could correctly identify the well-known relationship between the L-gulonolactone oxidase (*Gulo*) gene and vitamin C synthesis [50]. Therefore, we considered the same example, instead applying a dense feed-forward neural network for the prediction problem.

With this problem in mind, we designed GAP (Genotype-phenotype Association Predictor), a machine learning framework that employs a multi-layer neural network architecture to predict a binary phenotype solely from a multi-species sequence alignment. GAP has been implemented as an R package that is freely available along with a user guide and example dataset at https://github.com/uwaiseibna2/GAP. Its required input data consist of a multi-species sequence alignment from one or more genomic regions in any number of species, making it applicable to a wide range of biological problems and study systems. Additionally, GAP allows for the inclusion of a phylogenetic tree as input, which may help refine predictions by accounting for known evolutionary relationships among species.

## Methods

### Construction of the GAP neural network

**Figure 1:**
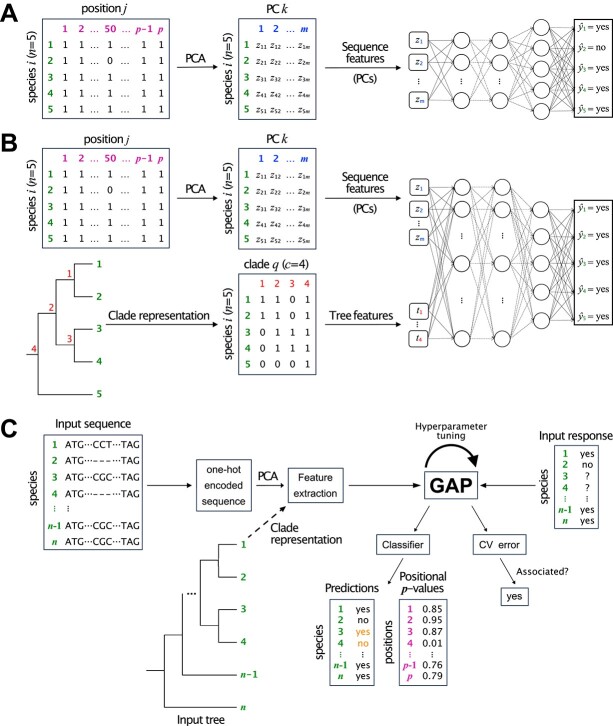
Schematic of the two approaches and general workflow of GAP. (A) The first approach takes as input a multiple alignment of a genomic region in $n$ species. This alignment is converted into matrix $\mathbf{x}$, with $n$ rows corresponding to species and $p$ columns corresponding to one-hot encoded positions, where position $j=0$ for a gap and $j=1$ otherwise. PCA is then applied to obtain matrix $\mathbf{z}$, with $n$ rows corresponding to species and $m<p$ columns corresponding to PCs that capture at least $95\%$ of the variation in $\mathbf{x}$. Last, $\mathbf{z}$ is used as input to an elastic net-penalized neural network with L $\in $ {0, 1, 2, 3} hidden layers. The output contains a vector of predicted class labels $\hat{y}_{i}$ for each $i \in \{1, 2, \ldots , n\}$ species. (B) The second approach takes as input a multiple alignment of a genomic region in $n$ species, as well as a tree relating the $n$ species. The multiple alignment is processed in the same way as for the first approach, generating matrix $\mathbf{z}$. Clades in the tree are represented as matrix $\mathbf{t}$ of 0s and 1s, where 0 and 1 denote the absence or presence of a species in clade $q$, respectively. Last, $\mathbf{z}$ and $\mathbf{t}$ are both fed as input into an elastic net-penalized neural network with L $\in $ {0, 1, 2, 3} hidden layers. The output contains a vector of predicted class labels $\hat{y}_{i}$ for each $i \in \{1, 2, \ldots , n\}$ species. In this example with $n=5$ species, the two approaches generate different predictions for species 2. (C) Both approaches utilize the same workflow, whereby an alignment and set of response values in $n$ species are provided as input, as well as a tree relating the $n$ species for the second approach. The alignment is one-hot encoded into matrix $\mathbf{x}$ and undergoes PCA to generate matrix $\mathbf{z}$, and the input tree is coded into matrix $\mathbf{t}$ if using the second approach. GAP then extracts $m<p$ PCs that capture at least $95\%$ of the variation in $\mathbf{x}$, as well as informative columns of $\mathbf{t}$ (columns 1–3) if using the second approach, which are provided as input features to the GAP neural network. Hyperparameter tuning is performed through elastic net regularization, yielding the trained classifier and cross-validation error. The trained classifier is used to generate an output file containing predicted phenotype class labels for each species with unknown status (orange text), as well as an output file containing $P$-values that denote the importance of each alignment position in the model. The cross-validation error is used to generate an output file containing information about whether the genomic region in the sequence alignment is putatively associated with the phenotype.

The goal of our study was to design a neural network that can predict a binary phenotype from gaps in a multi-species sequence alignment of one or more genomic regions. In particular, we developed a neural network to predict response $y$ with $K=2$ class labels ‘yes’ and ‘no’ denoting the presence or absence of a phenotype from gaps in a multi-species alignment of a genomic region (Fig. 1A). Therefore, for each genomic region, we first extracted information in species $i \in \{1, 2, \ldots , n\}$ about the presence (1) or absence (0) of a nucleotide at position $j \in \{1, 2, \ldots , p\}$ of the alignment as $x_{ij} \in \{0,1\}$, such that the vector of presence and absence information in species $i$ is given by


\begin{align*}& \mathbf{x}_{i} = (x_{i1}, x_{i2}, \ldots, x_{ip}) \in \mathbb{R}^{p}. \end{align*}

To reduce dimensionality of the data and account for correlations among genomic positions, we applied principal component analysis (PCA) and obtained the input feature vector of principal components (PCs) for species $i$ as


\begin{align*}& \mathbf{z}_{i} = \big(z_{i1}, z_{i2}, \ldots, z_{im}) \in \mathbb{R}^{m}, \end{align*}

where $z_{ij}$ is the projection of $\mathbf{x}_{i}$ onto principal component $k \in \{1,2,\ldots ,m\}$, such that the number of principle components (PCs) $m < p$ captures at least $95\%$ of the variation in the sequence alignment. Last, we trained an elastic net-penalized neural network with $L \in \{0, 1, 2, 3\}$ hidden layers to predict $y$ from $\mathbf{z}$, using cross-validation for model selection.

To account for evolutionary relationships among species, we also considered an extension of our initial approach that includes features describing the phylogenetic tree structure (Fig. 1B). Specifically, we obtained information about the presence or absence of each species $i \in \{1, 2, \ldots , n\}$ in each clade $q \in \{1, 2, \ldots , c\}$ of the phylogenetic tree as $t_{iq} \in \{0,1\}$, such that the vector of phylogenetic tree features in species $i$ is given by


\begin{align*}& \mathbf{t}_{i} = (t_{i1}, t_{i2}, \ldots, t_{ic}) \in \mathbb{R}^{c}. \end{align*}

These tree features $\mathbf{t}$ were used along with $\mathbf{z}$ to train an elastic net-penalized neural network with $L \in \{0, 1, 2, 3\}$ hidden layers to predict $y$, using cross-validation for model selection.

We used the ANN2 package [51] in R [52] to train each model. In particular, we considered a dense feed-forward neural network with $L \in \{0, 1, 2, 3\}$ hidden layers, in which each hidden layer has $k \in \{1, 2, \dots , n\}$ hidden units, where $n$ is the number of species. When $L=0$, the neural network simplifies to a linear model with logistic regression [53]. We denoted the input layer as hidden layer zero with $p=m$ features for the first prediction problem using $m$ PCs from the sequence alignment, and with $p=m+t$ features for the second prediction problem using the $m$ PCs along with $t$ tree features. Similarly, we denoted the output layer as hidden layer $L+1$ with $K=2$ class labels ‘yes’ and ‘no’. We applied linear and softmax activation functions [54] to the units in the input and output layer, respectively, and the rectified linear unit ($ReLU$; [54]) activation function defined as $ReLU$(x)= max(0, x) to units in hidden layers.

For each species $i$  $\in $ {1, 2, $\dots $, $n$}, the true class was denoted as $y_{i}$, and the predicted class as $\hat{y}_{i}$. The learning objective was to minimize the difference between $y_{i}$ and $\hat{y}_{i}$. We used binary cross-entropy deviance as the loss function to minimize the difference between true values and predicted probabilities [54]. To prevent overfitting, we employed elastic net regularization [55]. Specifically, we reduced the complexity of the fitted model with the tuning parameter $\lambda \geq 0$, which shrinks weights to zero, and the tuning parameter $\gamma \in [0,1]$, which determines the influence of $L_{1}$- and $L_{2}$-norm penalties for regularization [55]. We considered 100 values of $\lambda $ chosen uniformly across $\textrm{log} _{10}(\lambda ) \in [-4,3]$ and 20 values of $\gamma \in \{0, 0.05, \dots , 1\}$. We estimated model parameters from a number of hidden layers $L$ conditional on the pair of regularization tuning parameters $\lambda $, and $\gamma $. We used the Adam optimizer [56] with a learning rate of $10^{-2}$ and exponential decay rates for the first and second momentum of $\beta _{1}=0.9$ and $\beta _{2}=0.999$, respectively.

To estimate $L$, $\lambda $, and $\gamma $, we employed cross-validation [53]. In particular, because $n=34$ species in the empirical dataset on which we applied GAP, we used $n$-fold cross-validation, as it is well-suited for scenarios with limited observations [57]. However, we also included the option to perform $k$-fold cross-validation within our software. We employed mini-batch optimization with a batch size of 33 for 500 epochs to train the model. Cross-validation error was computed as the number of misclassified species divided by $n$, where a cross-validation error of zero indicates that the model made correct predictions for all $n$ species.

There are three main usages of GAP (Fig. 1C). First, given a known association between a particular genomic region and binary phenotype, one can make predictions about the presence or absence of the phenotype in species with unknown status. In this study, we made these predictions by applying our trained model selected through cross-validation to input features from the *Gulo* gene in 25 species with unknown vitamin C synthesis. Second, also given a known association between a genomic region and binary phenotype, one can pinpoint individual positions putatively associated with the phenotype. In particular, here positions in the *Gulo* gene with statistically significant $p$-values in our model were considered to be putatively associated with vitamin C synthesis due to their predictive importance. Third, in the scenario of an unknown association between a genomic region(s) and binary phenotype, one can apply GAP to multiple genomic regions to identify a set of candidates associated with the phenotype. Specifically, here we designated genes with the minimum cross-validation error among all genes as candidate genes, as they were able to most accurately predict vitamin C synthesis.

### Design of alternative machine learning architectures

For comparison with the GAP neural network, we also implemented Random Forest (RF), support vector machine (SVM), and extreme gradient boosting (XGB) architectures with the ranger [58], e1071 [59], and xgboost [60] packages in R [52], respectively.

For the RF, we used Breiman’s algorithm [61] with $n=500$ trees. To construct each tree, we generated a bootstrap training set of 58 observations through random sampling with replacement. For each split in a tree, a subset of size $q=\sqrt{p}$ of the features was chosen uniformly at random [58], and we split the node on one of these $q$ features by minimizing node impurity calculated with the Gini index [62]. We grew each tree without pruning [61], with a minimum node size of 10. Each of the $500$ trees in the RF contains estimated class probabilities [63], with the predicted class corresponding to the class with the larger mean estimated probability across all trees [61].

For the SVM, we employed a radial basis function (RBF) kernel [53] with 11 $\gamma \in \{0.001, 5\}$ hyperparameters chosen uniformly on a logarithmic scale. The benefit of using the RBF is that it is nonlinear but behaves as linear when $\gamma $ is small [53], enabling us to capture both linear and nonlinear relationships as with the neural network. The SVM uses this kernel to transform the feature space and identify the maximum margin hyperplane, which results in optimal separation of classes [64]. The predicted class is based on the sign of $y$, which indicates which side of the hyperplane the observation lies.

For the XGB, we explored a range of hyperparameters to optimize performance through cross-validation. Specifically, we tuned the model with a grid search over the number of boosting rounds $b$  $\in $ {50, 100}, maximum tree depth $d$  $\in $ [3, 7], learning rates $\eta $  $\in $ {0.01, 0.1}, and minimum loss reduction $\gamma $  $\in $ {0.0, 0.1} respectively. Additionally, we varied the fraction of features to be randomly sampled for each tree between $f_{p} \in $ {0.7, 0.8}, and the fraction of the training data to be randomly sampled for each boosting round $f_{t} \in $ {0.7, 0.8}. The output of XGB contains class probabilities, with the predicted class chosen as the class with the larger probability.

### Generation of empirical and simulated datasets

We used both empirical and simulated datasets for evaluation of the GAP neural network and three alternative machine learning architectures. For our empirical dataset, we took direction from [49], who developed a forward genomics approach to predict the ability to synthesize vitamin C from gaps in an alignment of the *Gulo* gene in 27 vertebrates. In particular, we chose vitamin C synthesis as the phenotype for our prediction task, but expanded our analysis to include input from aligned coding sequences of all 22,476 orthologous genes in 59 vertebrate species. Among these species, 18 are known to synthesize vitamin C (class ‘yes’), 16 are known to not synthesize vitamin C (class ‘no’), and 25 have unknown vitamin C synthesizing capabilities. Hence, the 34 species with known class labels were used for training, and the remaining 25 species were used as an empirical test set on which we later applied trained models to predict vitamin C synthesis.

Multiple alignments for exons of 22,476 protein-coding genes in 60 vertebrate species were downloaded from the UCSC Genome Browser at https://genome.ucsc.edu/. We excluded lamprey from our analysis due to the absence of the *Gulo* alignment despite knowledge of its presence in this species [65]. A literature search revealed that, of the remaining species, 18 can synthesize vitamin C (class ‘yes’; mouse, rabbit, rat, squirrel, tree shrew, mouse lemur, bushbaby, cow, dog, horse, cat, elephant, sheep, shrew, pig, chicken, wallaby, and platypus), 16 cannot synthesize vitamin C (class ‘no’; Guinea pig, marmoset, gorilla, human, gibbon, chimp, orangutan, Chinese rhesus, squirrel monkey, tarsier, microbat, megabat, zebrafish, Atlantic cod, medaka, and Nile tilapia), and 25 have unknown abilities to synthesize vitamin C [49, 50, 66–68]. Because we were interested in learning genotype–phenotype associations solely from alignment gaps, aligned exons of the 59 species were concatenated by gene and one-hot encoded, such that 0s represent gaps and 1s represent nucleotides. Non-variable alignment positions were removed, and the remaining positions were centered.

We extracted tree features for our second approach from the maximum likelihood species phylogeny estimated with IQ-TREE version 2 [69], which includes $c=58$ clades and matches the topology of the phylogeny found on the NCBI Taxonomy Browser [70, 71] at https://www.ncbi.nlm.nih.gov/Taxonomy/CommonTree/wwwcmt.cgi. IQ-TREE was chosen due to its widespread usage, better performance than other popular approaches, and efficiency [69, 72]. Moreover, it has a package for simulating sequences from gene trees, which we used to generate sequences for our simulation studies. For each clade, we assigned 1s to all species within the clade and 0s to all species outside the clade. Because the clade at the root of the tree contained all species, and therefore 1s for all species, it was uninformative and removed from our analysis. Therefore, we obtained $t=c-1=57$ tree features from this phylogeny, resulting in $p=m+57$ total input features for our second approach.

To generate our simulated dataset, we first used the tool hybrid-Lambda [73] to simulate 1,000 gene trees from the empirical species tree. Next, we used ALiSim [74] with the GTR+F substitution model determined by ModelFinder [75] to generate a sequence alignment with insertions and deletions for each simulated tree. Specifically, we set the mean length of simulated sequences to 1,575 nucleotides to reflect the mean length of genes in the empirical dataset and the indel rate to 0.23 to mimic that of *Gulo* [76]. We then used the sim.char method of geiger [77] to simulate an associated binary trait for each sequence alignment. To account for gene duplications and deletions, we used their distributions across all mouse genes and across all species to replace a similar subset of simulated sequences in each alignment with all gaps.

### Analysis of empirical and simulated data

We applied our trained GAP neural network, RF, SVM, and XGB architectures to the same empirical and simulated datasets. Empirical sequence alignments, either with or without the corresponding species tree, were used to predict vitamin C synthesis. To assess the importance of *Gulo* positions in predicting vitamin C synthesis, we reconstructed their coefficients from the $m$ PCs utilized for model training. In particular, model weights associated with $m$ principal components were multiplied by the matrix of loaded eigenvectors, yielding weights for each of the 1323 positions in *Gulo*. Subsequently, following [78], we calculated the squared Mahalanobis distance [79] between the coefficient of each position and the mean of the coefficients across positions. The squared Mahalanobis distance is distributed as a Hotelling’s $T$-squared distribution [80, 81] with test statistic $F$, enabling us to compute a $p$-value for each of the 1,323 *Gulo* positions. To account for multiple testing, we adjusted each $p$-value by employing the Benjamini-Hochberg procedure [82].

We performed Gene Ontology (GO) enrichment analyses to assess functional enrichment in genes identified by GAP with the web-based GOrilla tool [83, 84] at https://cbl-gorilla.cs.technion.ac.il/. In particular, we chose ‘*Mus musculus*’ as the organism, set the running mode to ‘Two unranked lists of genes (target and background lists)’, selected all ontologies (process, function, and component), and set the $P$-value threshold as $p=10^{-3}$. We uploaded the list of genes identified by GAP as the target list, and the list of all other genes in the mouse genome as the background list. To account for multiple testing, we only considered terms as significantly enriched if their false discovery rate (FDR) $q\text{-value} < 0.05$.

Simulated sequence alignments and associated traits were used solely or coupled with their corresponding species tree as input for GAP and the three alternative machine learning architectures. In particular, we first applied each method to each gene to predict its associated phenotype in each species and compute cross-validation error, and then to all 1000 genes to predict the gene(s) associated with a particular simulated phenotype and calculate cross-validation error rates, true positive rates (TPR, power), and false positive rates (FPR). We performed two-tailed two-sample permutation tests with the permTS function in the perm R package [85] to compare distributions of cross-validation errors between GAP and each of the alternative methods. Resulting $p$-values were Bonferroni-corrected to account for the three comparisons performed.

## Results

### Evaluation of GAP performance

We first applied GAP to the *Gulo* gene alignment with and without the species tree to predict vitamin C synthesis. These empirical analyses both yielded cross-validation errors of zero for several neural network architectures (Tables S1 and S2). Hence, we obtained perfect accuracy for predicting the known association between *Gulo* and vitamin C synthesis [50, 86] with models of varying complexities, regardless of whether we included tree features as input. Of these models, we selected the simplest model, i.e., the model with the minimum number of hidden layers yielding the smallest cross-validation error. For this example, the simplest model contained zero hidden layers for each input dataset. Obtaining perfect accuracy with a fairly simple model is of course an ideal scenario, and it is not expected that all applications of GAP will produce identical results. We initially hypothesized that this finding reflects the ease of predicting the well-known relationship between *Gulo* and vitamin C synthesis, perhaps due to the presence of a clear evolutionary signature in the *Gulo* alignment. However, we were unable to achieve perfect accuracy with any of the three alternative machine learning architectures, either with or without the species tree included as input (Tables S1 and S2), demonstrating that the GAP neural network is ideal for this particular prediction problem.

To more rigorously evaluate the performance of GAP, we next applied it to each of the 1,000 simulated sequence alignments with and without the species tree as input to predict the associated phenotype in each species. These analyses resulted in mean cross-validation errors of 0.043 when using solely the sequence alignment as input and 0.052 when including the species tree, both of which were significantly lower than those of all three alternative machine learning architectures (Tables S3 and S4). We then set a cross-validation error threshold of 0.1, assigned random binary phenotypes not associated with the simulated sequence alignments, and applied GAP with and without the species tree as input to estimate TPR (power) and FPR. We found that GAP tended to correctly identify the associated gene, with TPRs of 0.915 when using solely the sequence alignment as input and 0.855 when including the species tree, both of which were again higher than those of all three alternative machine learning architectures (Tables S3 and S4). Moreover, GAP generally did not falsely identify genes that were not associated with the phenotype, with FPRs of 0.117 when using solely the sequence alignment as input and 0.067 when including the species tree, whereas all three alternative machine learning architectures had FPRs of zero for both sets of input data (Tables S3 and S4). Though TPR and FPR are positively associated, and we therefore expected small FPRs for the three alternative architectures, it is interesting that they were zero. We believe that this result stems from the conservativeness of these architectures, which have much harder decision boundaries than a neural network. Collectively, our simulation results demonstrate that GAP demonstrates excellent performance overall, achieving higher accuracy and sensitivity with a tradeoff of slightly less specificity relative to three alternative machine learning architectures. Further, comparisons of performance metrics between GAP input datasets show that including the species tree decreases accuracy and true positive rates, but also decreases false positive rates, indicating that it may be useful for refining predictions in some scenarios.

### Predicting a phenotype in species with unknown status

**Figure 2:**
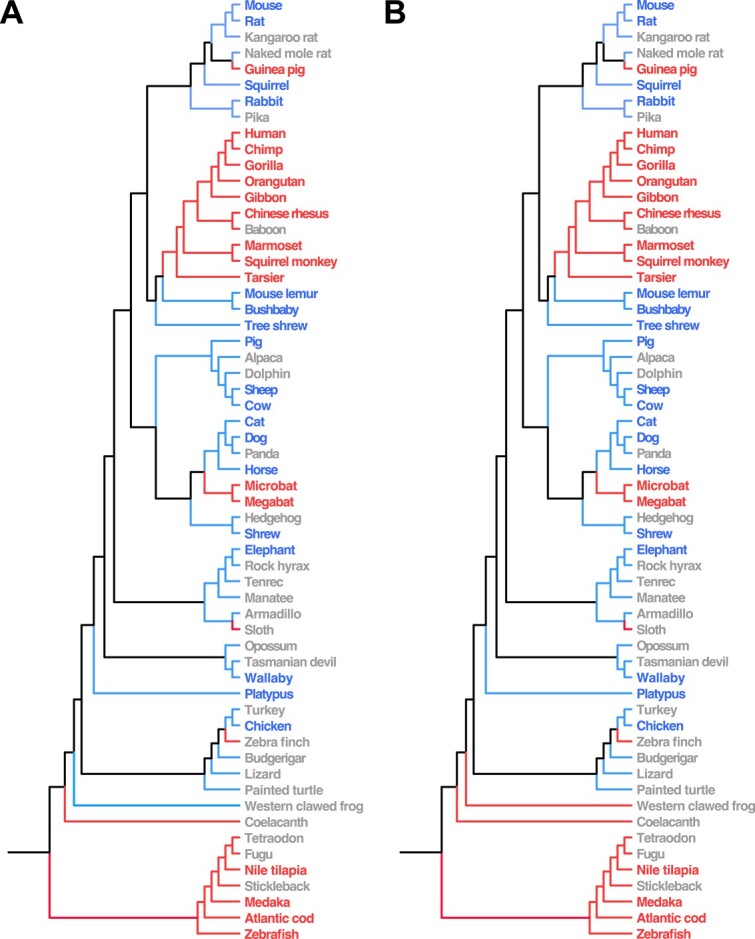
Known and predicted vitamin C synthesis for 59 species depicted on their phylogenetic tree. Species names are colored to denote vitamin C synthesis determined by prior studies: blue for class ‘yes’, red for class ‘no’, and gray for unknown. Branches are colored to denote vitamin C synthesis predicted by GAP: blue for class ‘yes’ and red for class ‘no’. Predictions are shown for the approaches based on input features from the alignment only (A), and input features from the alignment and phylogenetic tree (B).

The first problem that we sought to address in our study was predicting a binary phenotype in one or more species with unknown status. Therefore, we applied our two approaches (Fig. 1) to input features from the *Gulo* alignment to predict vitamin C synthesis in the 25 vertebrates with unknown status. Because we obtained cross-validation errors of zero for both approaches, all predicted class labels match those of species with known vitamin C synthesis (Fig. 2). Additionally, predicted class labels for the 25 species with unknown vitamin C synthesizing status are the same for both approaches, with the exception of western clawed frog. Application of the alignment-only approach predicts that this species synthesizes vitamin C, whereas including tree features predicts the opposite. To better understand this discordance, we examined the *Gulo* sequence of western clawed frog. We found that approximately 24% of the *Gulo* sequence is missing in this species, which is not atypical of species that are known to synthesize vitamin C. It is also notable that western clawed frog (an amphibian) is an outgroup to a clade containing another amphibian (painted turtle), a reptile (lizard), and several birds. Given these findings, it is understandable how GAP may have difficulty with predicting the vitamin C synthesis in western clawed frog, and, therefore, why its predictions vary based on whether the phylogenetic tree is provided as input.

Considering the remaining 24 species with the same predictions for both approaches, 21 have predicted class labels that match the known or predicted class labels of their closest relatives. Hence, predictions are generally consistent with phylogenetic relationships among species, regardless of whether the tree is provided as input. The three exceptions are naked mole rat, sloth, and zebra finch. Naked mole rat is predicted to synthesize vitamin C, though its closest relative guinea pig is known not to synthesize vitamin C [50]. On the other hand, both sloth and zebra finch are predicted to not synthesize vitamin C, contrasting with the predictions of their closest relatives armadillo and budgerigar, respectively. To determine why our predicted class labels for three species do not match those known or predicted in their closest relatives, we also considered other related species with known vitamin C synthesis. For naked mole rat, we examined the seven other rodents in our dataset: mouse, rat, kangaroo rat, guinea pig, squirrel, rabbit, and pika. Among these, four species are known to synthesize vitamin C (mouse, rat, squirrel, and rabbit), whereas the only species that is known to not synthesize vitamin C is guinea pig. Therefore, our prediction of vitamin C synthesis for naked mole rat is consistent with the known class labels of most rodents. However, this same type of comparison cannot be performed for sloth, as among the five other species of Atlantogenata (elephant, rock hyrax, tenrec, manatee, and armadillo), only elephant has a known class label that is inconsistent with the loss of vitamin C synthesis predicted for sloth. Similarly, there are three bird species aside from zebra finch (turkey, chicken, and budgerigar), and only chicken has a known class label that conflicts with our prediction of a loss of vitamin C synthesis in zebra finch. Examination of the *Gulo* alignment, however, supports all three of these predictions. In particular, there are no gaps in the alignment for naked mole rat, supporting our prediction of vitamin C synthesis in this species. In contrast, over 50% of the *Gulo* sequence is missing in sloth, including eight full exons and one partial exon, and the entire sequence is missing in zebra finch, consistent with losses of vitamin C synthesis in both species. Therefore, our predictions for naked mole rat, sloth, and zebra finch align well with expectations based on prior studies of the association between deletions of *Gulo* sequences and loss of vitamin C synthesis [49, 50].

### Pinpointing genomic positions associated with a phenotype

**Figure 3:**
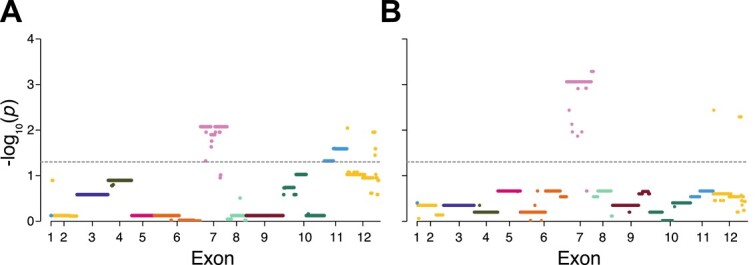
Manhattan plots depicting distributions of $p$-values across *Gulo* exons. Points are colored by exon, values along $x$ axes represent center positions of exons, and horizontal gray dashed lines denote the $p$-value significance cutoff of $\alpha = 0.05$ after Benjamini–Hochberg adjustment. Manhattan plots are shown for the approaches based on input features from the alignment only (A), and input features from the alignment and phylogenetic tree (B).

Next, we considered the task of identifying specific genomic positions that are associated with a binary phenotype of interest. As there is a known relationship between *Gulo* and vitamin C synthesis [50], and we obtained perfect accuracy in predicting this relationship in species with known vitamin C synthesis, we considered the importance of individual positions in our predictive model. This strategy has been employed in prior studies to enhance interpretability of the underlying model by uncovering associations at the molecular level [87, 88]. To visualize the predictive importance of individual positions, we constructed Manhattan plots showing distributions of their $p$-values across *Gulo* exons (Fig. 3). For both approaches, neighboring positions tend to have similar levels of importance, often with clear boundaries between large segments that include exons. However, there are differences in *Gulo* positions predicted to be associated with vitamin C synthesis between the two approaches. Positions in three exons (7, 11, and 12) are statistically significant with the approach based on only alignment features, whereas positions in two of these exons (7 and 12) remain significant when tree features are included in the prediction problem (Fig. 3). Intriguingly, this difference can be used to explain the discrepancy in predictions between approaches for western clawed frog, as exons 11 and 12 are both absent in this species.

To better understand the associations of positions in exons 7, 11, and 12 to vitamin C synthesis, we compared their sequences between vitamin C-synthesizing and non-synthesizing species. We considered species with predicted class label ‘yes’ for both approaches as vitamin C-synthesizing, and those with predicted class label ‘no’ for both approaches as non-synthesizing (Fig. 2). Thus, due to conflicting predictions of the two approaches, western clawed frog was not used for this analysis. For exon 7, mean percentages of gaps were 8.9% for vitamin C-synthesizing species and 49.8% for non-synthesizing species. For exon 11, mean percentages of gaps were 0% for vitamin C-synthesizing species and 74.2% for non-synthesizing species. For exon 12, mean percentages of gaps were 1.6% for vitamin C-synthesizing species and 40.7% for non-synthesizing species. Thus, there are large differences between classes for these exons, and particularly for exon 11, which has no gaps in vitamin C synthesizing species and is missing most of its sequence in non-synthesizing species. These findings are generally consistent with prior studies, which have shown that exons 7 and 11 diverge the most between vitamin C-synthesizing and non-synthesizing species [89], and that exon 12 has been partially or fully lost in bats and some primates that cannot synthesize vitamin C [50, 89, 90].

### Identifying candidate genomic regions associated with a phenotype

Last, we addressed the problem of identifying genomic region(s) associated with a binary phenotype of interest. Thus, we applied GAP to alignments of 22,476 mouse genes to identify putative candidate genes associated with vitamin C synthesis. In addition to *Gulo*, we identified 3,034 genes with the approach based only on alignment features (Table S5) and 1,536 genes when including tree features (Table S6). Thus, approximately $13.5\%$ or $6.8\%$ of mouse genes are predicted to be associated with vitamin C synthesis with the alignment-only and tree-based approaches, respectively. These large proportions may be attributed to gains and losses of many unrelated genes throughout vertebrate evolution. Indeed, 811 genes for the alignment-only approach and 602 genes for the tree-based approach are olfactory genes, which underwent large expansions in rodent lineages [91, 92].

To assess the functions of genes predicted to be associated with vitamin C, we identified Gene Ontolog (GO) [93] terms enriched in these genes relative to the genome-wide background in mouse. This analysis yielded numerous significant GO terms for each approach (Tables S7-S12), many of which are unsurprisingly related to olfactory receptor activity, but with another large subset corresponding to immunity. Closer inspection of GO terms uncovered 241 and 145 immunity-associated genes for the alignment-only and tree-based approaches, respectively. Hence, this analysis substantially narrowed the initially large lists of candidate genes from the alignment-only and tree-based approaches down so that they represent approximately $1.1\%$ and $0.6\%$ of mouse genes, respectively. Many of these genes are from the interferon, chemokine, histocompatibility agent, defensin, $cd$ antigen, interleukin, and eosinophil-associated ribonuclease (*mEAR*) families. Genes identified by this analysis are therefore consistent with the phenotype under consideration, as vitamin C contributes to immune defense by supporting various cellular functions of both the innate and adaptive immune system [94–97]. Thus, these findings demonstrate that GAP can provide a fruitful list of candidate genes for downstream analyses.

## Discussion

In this study, we presented GAP, a novel machine learning framework for predicting a binary phenotype from gaps in a multi-species sequence alignment. GAP implements an elastic net-regularized neural network architecture and can be employed for three distinct problems: predicting a phenotype in species from a known associated genomic region, pinpointing specific positions within such a genomic region that are important for predicting a phenotype, and identifying candidate genomic regions that may be associated with a phenotype. We illustrated that, despite its relatively simple framework, GAP demonstrates exceptional performance on simulated data. Moreover, application of GAP to a multi-species sequence alignment of the *Gulo* gene from 59 vertebrates showed that it can predict the known relationship between *Gulo* and vitamin C synthesis with perfect accuracy, that its predictions of vitamin C synthesis in species with unknown status closely match their phylogenetic relationships, and that the positions it assigns as important are generally consistent with previous findings [50, 89, 90]. Further, a genome-wide application of GAP uncovers many additional genes putatively associated with vitamin C synthesis, and these candidates are enriched for immunity functions similar to those of vitamin C. Thus, our study demonstrates the utility of GAP in addressing multiple fundamental questions about genotype–phenotype associations from data that are widely available for many species.

Though GAP can make predictions solely from a multi-species sequence alignment, it also allows for an optional phylogenetic tree input. Our simulation analyses show that both approaches can be useful, with the alignment-only approach yielding higher accuracy and power, and the tree-based approach resulting in lower false positive rates and therefore better refinement of predicted genomic regions. Consistent with these findings, accounting for phylogenetic tree structure did not improve performance in our case study with the *Gulo* gene—perfect accuracy was achieved regardless of the approach employed. However, this may not be the case for all applications. In particular, vitamin C synthesis has been independently lost many times throughout vertebrate evolution [50], and information gleaned from phylogenetic relationships may be less useful in such scenarios. Indeed, using only species tree features within the same framework resulted in a minimum cross-validation error of $0.04$, indicating that there is some other information in the alignment that aids in predicting vitamin C synthesis. We hypothesize that this missing information comes from the gene tree. Yet we obtained minimum cross-validation errors of 0.058 when using solely gene tree features, and 0.088 when using gene tree and species tree features together. Hence, the alignment (with or without a phylogeny) is required for perfect accuracy, perhaps because it contains hidden information about the contributions of gene tree and species tree evolution to the loss of vitamin C synthesis. In particular, it is likely that GAP performs well when applied solely to the sequence alignment because it learns how to optimally place importance on characteristics of the species tree and gene tree that are embedded within the alignment. On the other hand, inclusion of a phylogenetic tree may help refine predictions, as accounting for species relationships led to lower false positive rates in our simulations and fewer identified positions and candidate genes associated with vitamin C synthesis. For this reason, we chose to include the phylogenetic tree as an optional input to the GAP software package.

For the approach incorporating phylogenetic tree structure, we would also like to note that the number of tree features increases rapidly with the number of species, which can lead to greater emphasis being placed on phylogenetic tree structure by our second approach. Thus, when the number of species under consideration is large or the species tree is unresolved, it may be best to either employ the sequence-only approach or utilize both approaches and then carefully compare findings when drawing conclusions. Additionally, our software implementation of GAP provides an option to perform five-fold rather than $n$-fold cross-validation, which substantially speeds up computation time for model selection and is recommended when the number of species exceeds 50 and resources are limited.

Deciphering the evolutionary dynamics of phenotype gains and losses is a significant frontier, and thus a lot of legwork will likely be needed to investigate and validate the predictions output by GAP. Nevertheless, we believe that GAP represents a valuable tool for predicting genotype–phenotype associations, particularly when one has access to limited data. Though more complex prediction algorithms that incorporate a diversity of contemporary data types are of course welcome, such methods may have limited utility in the short-term. In contrast, GAP can be used with many datasets that are either publicly available or relatively easy and cheap to produce in modern times. Input sequence alignments are provided to GAP in FASTA format and optional phylogenetic trees in Newick format, both of which are the most common formats found in public databases. GAP then automatically performs all necessary conversions, including one-hot encoding of the sequence alignment and extraction of both alignment and tree features. Behind the scenes, GAP also adopts parallelization for computational efficiency, making it both easy to use and scalable.

The algorithm implemented by GAP was designed to maximize computational efficiency through dimensionality reduction with PCA. Though this approach is generally effective, as it captures a majority of the variation in the input data, it is possible that an important feature will be excluded. In a similar vein, we chose to base predictions solely on gaps in the input sequence alignment. Though losses of gene function commonly occur through insertions and deletions [6, 47, 48], including in *Gulo* [50], they can also be attributed to nucleotide substitutions or regulatory changes outside of the coding region of a gene [98, 99]. In the case of nucleotide substitutions, one can extend the GAP framework to take the original sequence as input via implementation of a convolutional neural network [100, 101]. However, accounting for regulatory changes is a more difficult problem due to the unknown influences of *cis*- and *trans*-acting elements on gene function [102–104]. In such cases, it may be useful to incorporate information from expression quantitative trait loci. Other future improvements to GAP include extending predictive models to non-binary phenotypes, exploring advanced algorithms, and integrating additional biological features to enhance cross-species phenotype predictions. In particular, the incorporation of multi-omics data spanning genomics, transcriptomics, and proteomics is a promising direction for a more comprehensive understanding of the genetic basis of phenotypic variation.

**Key Points**

- We introduce GAP, a novel framework for predicting binary phenotypes from gaps in multi-species sequence alignments.
- GAP can be used to predict a phenotype in species from a known associated genomic region, pinpoint specific positions within such a region that are important for predicting a phenotype, and extract a set of candidate genomic regions associated with a phenotype.
- Our study shows that GAP has excellent performance on simulated data, perfect accuracy in predicting the well-known association between the *Gulo* gene and vitamin C synthesis, and findings that are compatible with phylogenetic expectations and prior studies.

## Supplementary Material

IslamEtAl2024_SupplementaryTables_bbaf022

## Data Availability

The GAP software package, instructions, and associated input and output datasets are available at https://github.com/uwaiseibna2/GAP.
